# Novel Tissue Engineering Scaffolds in the Treatment of Spinal Cord Injury—A Bibliometric Study

**DOI:** 10.3390/bioengineering12040347

**Published:** 2025-03-28

**Authors:** Yan Zhao, Abudunaibi Aili, Zhiwei Jia, Tianlin Wen, Aikeremujiang Muheremu

**Affiliations:** 1 Department of Rehabilitative Medicine, Sixth Affiliated Hospital of Xinjiang Medical University, Urumqi 830063, China; zhaoyanlfy@163.com; 2 Key Laboratory of Orthopaedic Regenerative Medicine, Sixth Affiliated Hospital of Xinjiang Medical University, Urumqi 830063, China; muheremua@bjmu.edu.cn; 3 Department of Orthopaedics, Dongzhimen Hospital, Beijing University of Chinese Medicine, Beijing 100013, China; jiazhiweivip@126.com

**Keywords:** spinal cord injury, axonal regeneration, tissue engineering, hydrogels, cell transplantation, bibliographic study

## Abstract

Objective: Because of the evolving nature of tissue engineering scaffolds in the treatment of spinal cord injury (SCI), the current study was carried out to evaluate the research productivity of tissue engineering scaffolds in the treatment of SCI. Methods: Studies published from 2000 to 2025 were retrieved from the Web of Science core collection with topics of spinal cord injury and tissue engineering scaffolds. The data were analyzed and visualized using the VOSviewer network analysis software. Results: Among 1542 articles analyzed, annual publications surged from 2000 to 2019, stabilizing thereafter. The U.S., China, and Canada led in productivity, with Northwestern University and the *Biomaterials* journal being top contributors. Keyword analysis revealed research hotspots such as functional recovery, axonal regeneration, stem cells, and hydrogels. Notably, hydrogels embedded with genetically engineered cells emerged as a pivotal trend, reflecting a shift toward biomimetic and combinatorial therapies. Collaboration networks highlighted intensified partnerships between Chinese and North American institutions, signaling global interdisciplinary efforts. Conclusions: This study provides the first bibliometric roadmap for tissue engineering scaffolds in SCI, identifying key trends, influential entities, and underexplored areas. The rise in hydrogels and international collaborations underscores opportunities for targeted research. These findings guide researchers in prioritizing high-impact journals, fostering partnerships, and advancing novel scaffold designs to bridge translational gaps in SCI treatment.

## 1. Introduction

Spinal cord injury (SCI) is a devastating central nervous system disorder that frequently results in the partial or complete loss of sensory, autonomic, and motor functions, as well as impaired sphincter control [1,2]. Current clinical interventions, such as surgical stabilization, corticosteroid administration, and rehabilitative therapies, aim to mitigate secondary damage and preserve residual function. However, these approaches fail to address the core challenges of neural regeneration, including the hostile inflammatory microenvironment, inhibitory glial scar formation, and the absence of structural guidance for axonal regrowth. Even emerging cell-based therapies, though promising, grapple with limitations such as low cell survival rates, uncontrolled differentiation, and immune rejection. These shortcomings underscore the critical need for innovative strategies capable of reconstructing the spinal cord’s intricate architecture while dynamically modulating the injury milieu to support repair. In the realm of regenerative medicine, tissue engineering scaffolds have demonstrated significant potential in various studies [3,4]. These scaffolds can establish a conducive microenvironment for the survival of seed cells, bridge injury-induced defects, inhibit the formation of glial scars, provide essential guidance for axonal regeneration, limit local inflammatory responses, prevent apoptosis, and enhance axonal growth [5,6,7].

Despite these advances, the field remains fragmented, with disparate research efforts often lacking coordination or strategic focus. A systematic evaluation of the global research landscape is essential to identify trends, prioritize resources, and bridge translational gaps. However, forming a comprehensive assessment of this field remains challenging due to the labor-intensive and time-consuming nature of manual compilation. Traditional reviews, while valuable, tend to emphasize experimental outcomes rather than mapping the field’s intellectual structure or collaborative networks. Bibliometric analyses, which quantitatively assess publication patterns, authorship dynamics, and keyword evolution, offer a powerful yet underutilized tool to address this need [8,9]. To date, no study has applied bibliometric methods to evaluate tissue engineering scaffold research in SCI, leaving stakeholders without a consolidated roadmap to navigate this rapidly evolving domain.

In this study, we utilized VOSviewer software to conduct a bibliometric analysis of the literature on the application of tissue engineering materials in SCI treatment. As the inaugural bibliometric investigation in this domain, our study aims to delineate the global research trajectory, highlighting leading countries, institutions, and journals; identify emerging trends, such as hydrogel–cell composite therapies and nanotechnology-driven scaffolds; and uncover underexplored areas ripe for innovation. Our findings provide a strategic framework to guide future research, foster cross-disciplinary collaborations, and accelerate the translation of scaffold-based therapies from bench to bedside.

## 2. Materials and Methods

A systematic literature search was conducted in the Web of Science (WoS) Core Collection to identify peer-reviewed articles on tissue engineering scaffolds for spinal cord injury.

### 2.1. Search Strategy and Keyword Selection

To ensure a comprehensive and reproducible search, the Web of Science (WoS) Core Collection was queried using a systematic keyword strategy. The search terms were derived through a three-step process, including (1) concept identification: core concepts included “spinal cord injury” (SCI), “tissue engineering scaffolds”, and “biomaterials”. (2) Term expansion: Synonyms and related terms were identified through preliminary literature reviews and Medical Subject Headings (MeSH). For example, SCI variations included “spinal cord trauma” and “spinal cord lesion”; scaffold variations included “3D matrix”, “hydrogel”, and “polymer scaffold”; and biomaterial variations included “synthetic polymer”, “natural polymer”, and “decellularized matrix”. (3) Boolean logic: terms were combined using Boolean operators to maximize sensitivity and specificity, producing Topic Set 1, (“tissue engineering” OR “biomaterial*”) AND (“spinal cord injur*” OR “SCI”), and Topic Set 2, (“scaffold*” OR “hydrogel*” OR “3D matrix”) AND (“axonal regeneration” OR “neural repair”).

### 2.2. Database Search and Data Extraction

Timeframe: January 2000–January 2025. The start year (2000) was chosen to align with the emergence of modern tissue engineering techniques, while the end date (2025) reflects the study’s inclusion of early-access articles indexed ahead of print.

Document types: articles, reviews, and systematic reviews.

Inclusion and Exclusion Criteria

Inclusion: Peer-reviewed articles (2000–2025) addressing scaffold design, in vivo applications, or clinical trials for SCI. English-language publications. Studies integrating scaffolds with cells, genes, or bioactive molecules.

Exclusion: Editorials, conference abstracts, or non-English papers. Studies on peripheral nerve injury or non-scaffold-based therapies (e.g., pharmacologic interventions).

Search validation: A pilot test compared the recall and precision of keyword combinations. Final terms achieved >90% relevance in a random sample of 100 articles.

Data extraction: records were deduplicated using WoS’s native tools, followed by manual screening to remove irrelevant studies (e.g., non-SCI applications).

### 2.3. Bibliographic Analysis

A bibliographic network analysis was conducted using VOSviewer (version 1.6.14, Centre for Science and Technology Studies, Leiden University, Leiden, The Netherlands), a software tool specifically designed for constructing and visualizing bibliometric networks. Its clustering algorithms excel at disentangling complex co-occurrence relationships, enabling clear identification of keyword clusters and collaboration patterns. VOSviewer allows granular adjustments to node size, color gradients, and layout scaling, critical for mapping temporal trends and enhancing interpretability. The tool efficiently processes large datasets while maintaining resolution in visual outputs, ensuring a robust representation of high-dimensional data [10].

Attributes such as keywords, authors, institutions, and countries were examined. The term maps for software analysis were generated with the following settings: “create a map based on bibliographic data”, “read data from bibliographic database files”, “type of analysis: co-occurrence”, “unit of analysis: all keywords”, and “counting method: full counting”. The statistical analysis results were visualized as a network of circles, each representing an item. The size of each circle corresponded to the item’s frequency in various publications, while the color indicated its cluster. The distances between circles reflected the frequency of their co-occurrences. Country-specific data on related research were visualized using Excel 2019 (Microsoft, Redmond, WA, USA).

An annual publication analysis was carried out to assess the trends in the field of tissue engineering materials and spinal cord injury by visualizing annual scientific publications and average citation counts. The impact of countries, institutions, journals, and authors was evaluated based on bibliometric indicators such as annual publications and local/global citations. Additionally, collaborative relationships between countries and authors were evaluated, and country collaboration networks and author collaboration networks were constructed. The journals publishing the literature were analyzed to identify the most relevant sources and their influence. The most globally and locally cited documents were identified by cited documents and reference analysis, and a historical direct citation network map was constructed to illustrate citation relationships among documents. Research hotspots were analyzed through keyword co-occurrence networks and historical direct citation network maps. The keyword co-occurrence network demonstrated relationships between keywords, while the historical direct citation network map showed inheritance relationships among documents. Through these analytical methods, this study systematically reviewed the current status, hotspots, and trends in the field of apoptosis and spinal cord injury, providing important references and guidance for future research.

## 3. Results

A total of 1542 articles were included in the final analysis. Over the past 20 years, the annual number of publications in this field has consistently increased, peaking in 2019. Subsequently, the annual publication rate has stabilized. The top three funding sources are the National Natural Science Foundation of China (NSFC), the European Commission, and the National Institutes of Health (NIH).

### 3.1. Main Countries Contributing to This Field

**Figure 1:**
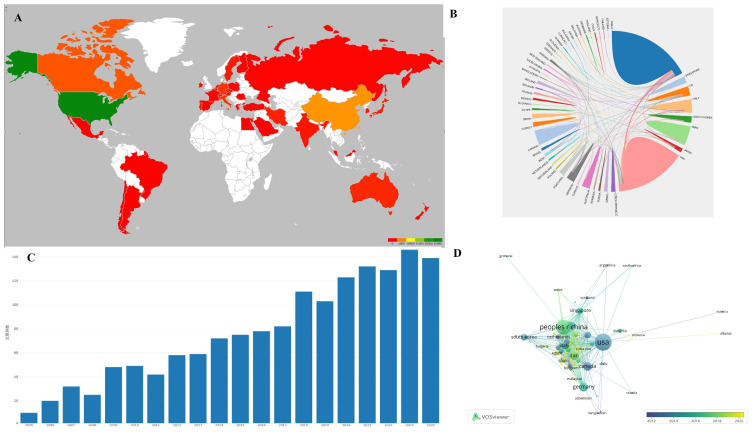
(**A**) Main countries contributing to the field; color reflects the number of publications per country, with green reflecting the country with the most publications and shallow green, yellow, orange, and red for countries with less papers. (**B**) Analysis of international collaboration. The size of each country’s segment in the circular diagram corresponds to its number of publications, while the thickness of the connecting lines indicates the strength of collaborative ties between countries. (**C**) Annual publication trends: steady growth from 2000 to 2019, plateauing post-2020 due to maturation of scaffold technologies. 文章总数 = total numbers of papers. (**D**) Visualization of citation networks among countries. Each country is depicted as a node, with the size of the node reflecting the volume of citations it has received (USA leads with 20,956 citations). Gradient colors (blue to yellow) show citation density over time.

VOSviewer software was utilized to create a visualization atlas of the co-occurrence of countries, as illustrated in Figure 1 and Table 1. The most cited papers on tissue engineering-based therapies for SCI primarily originate from the USA, followed by China, Canada, England, and Italy. The USA is the leading contributor in terms of citing articles. However, visualization analysis indicates that China has emerged as a significant contributor in this field in recent years.

### 3.2. Research Productivity of Institutions

**Figure 2:**
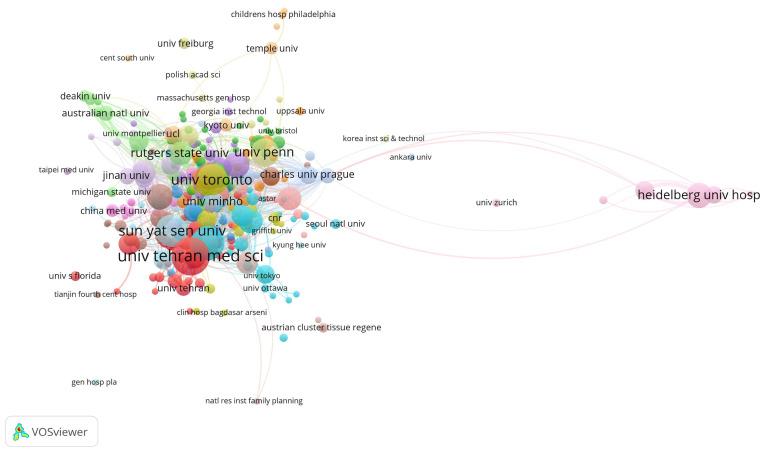
Main institutions contributing to research on tissue engineering-based treatment strategies for spinal cord injury. Each node represents an institution, with its size corresponding to the number of citations received. The node’s color denotes distinct research topics (red is biomaterials; blue is stem cells; and green is clinical translation), while the connecting lines between nodes signify co-citation relationships.

The top three institutions with the highest total citations are Northwestern University (2342), the University of Toronto (2146), and Case Western Reserve University (1652). The leading institutions in terms of publication volume are the University of Tehran Medical Sciences (36), Nantong University (30), and Sun Yat-sen University (27) (Figure 2, Table 2). Young researchers might consider these institutions when applying for studies or positions in this field.

### 3.3. Keyword Co-Occurrence

**Figure 3:**
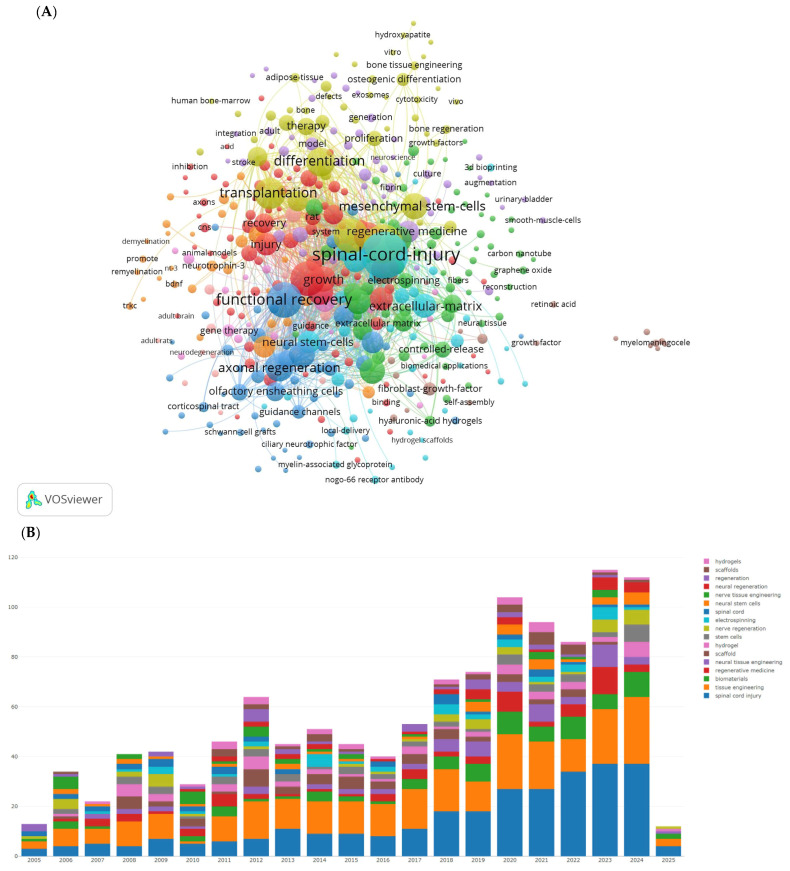
The most popular key words in studies on tissue engineering-based treatment strategies for spinal cord injury. (**A**) Visualization of the keyword co-occurrence network. Each node represents a keyword, with its size reflecting its frequency of occurrence. The node’s color corresponds to different research topics (red represents regeneration-focused terms such as axonal regeneration, functional recovery; blue represents stem cell and transplantation themes; and green represents biomaterial innovations such as hydrogels and the extracellular matrix), while the connecting lines between nodes indicate co-occurrence relationships. (**B**) The bars shows how the focus of research has shifted over time: In the early 2000s, there was a dominance of in vitro studies and differentiation. Post-2010, there has been a rise in research on hydrogels and genetic engineering.

We used VOSviewer software to display the visualization map of keywords, as shown in Figure 3 and Table 3. After removing SCI and tissue engineering scaffold nodes, the top ten keywords by frequency are functional recovery, in vitro, regeneration, transplantation, differentiation, axon regeneration, stem cells, repair, and neurotrophic factors. The most prominent keywords include rat SCI, tissue engineering scaffolds, adult rats, glial transplantation, recovery, mesenchymal stem cells, Schwann cells, extracellular matrix, and hydrogels. Hydrogels are a network of polymer chains that are commonly mentioned in a broad number of publications discussing the design of tissue engineering materials.

### 3.4. Journal Analysis

**Figure 4:**
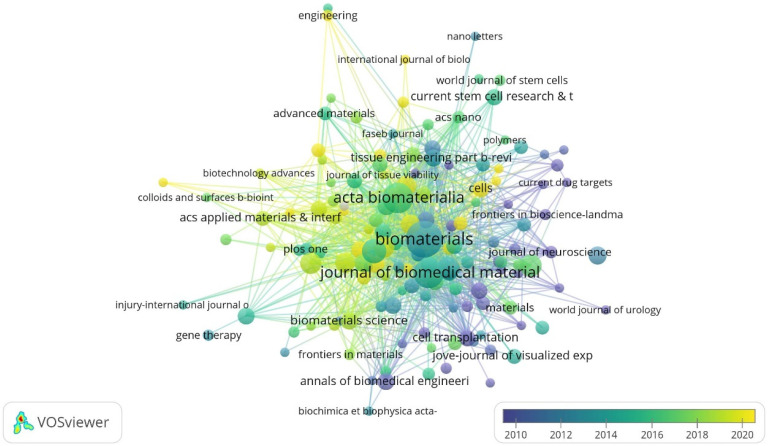
Citation overlay visualization for institutions. Each institution is depicted as a node, with the node size reflecting its citation count (*Biomaterials*: 4112 citations). The connecting lines between nodes are color-coded based on a gradient scale corresponding to the years (blue—pre-2010 period; yellow—post-2020 period).

The top journals with the largest number of citations in this field are *Biomaterials*, *Acta Biomaterialia*, *Journal of Biomedical Materials Research Part A*, *Neural Regeneration Research*, and *Tissue Engineering Part A*. The top three journals with the highest impact factor (IF) are *Advanced Functional Materials* (19.9), *Biomaterials* (15.3) and *Advanced Healthcare Materials* (11.1) (Figure 4, Table 4). This could serve as a reference for choosing the publishers of high-quality papers in this field.

### 3.5. Top 10 Most Productive Authors

**Figure 5:**
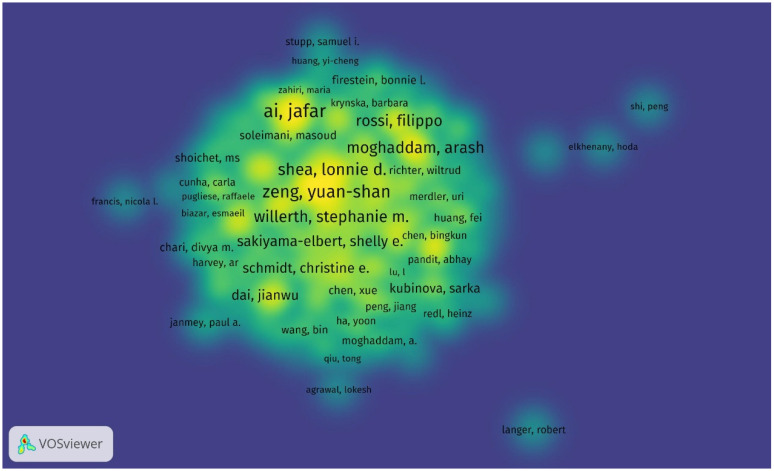
Density visualization of the most productive authors in research on tissue engineering-based treatment strategies for spinal cord injury shows that the leading authors like Stephanie M. Willerth (865 citations) anchor collaborative networks, while emerging researchers (e.g., Fabian Westhauser) show rising productivity. Color intensity indicates the author’s influence (bright yellow—most cited; blue—niche contributors). Overlay: collaborative clusters (e.g., Yuan-shan Zeng’s team forms a dense hub in China).

Throughout 2000–2025, the top five authors with the most cited publications in this field are Ai, Jafar; Zeng, Yuan-shan; Ebrahimi-Barough, Somayeh; Chew, Sing Yian; Shea, Lonnie D., and Willerth, Stephanie M., who is the top-cited author with a total of 865 cited articles and an average of 66 citations per publication. The top three cited authors are Willerth, Stephanie M. (865 times); Shea, Lonnie D. (768 times); and Chew, Sing Yian (687 times). The top three authors based on total link strength are Zeng, Yuan-shan; Chew, Sing Yian; and Zeng, Xiang (Figure 5, Table 5).

### 3.6. Top-Cited Publications in the Field

**Figure 6:**
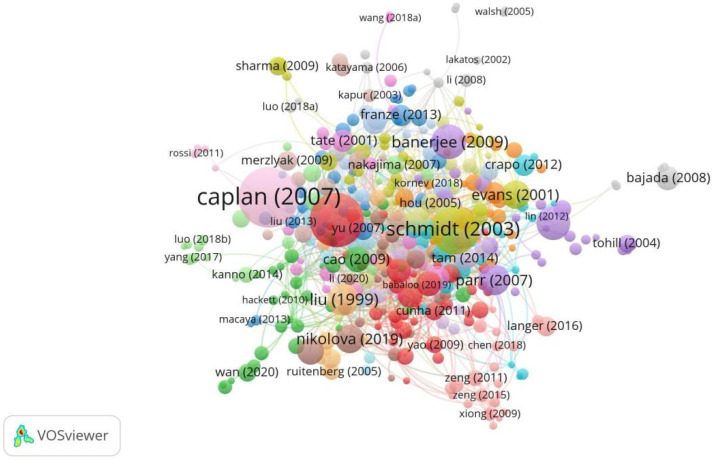
Top-cited publications in research on tissue engineering-based treatment strategies for spinal cord injury show that foundational works (Lundborg, 2000) [15] anchor the field, while newer studies (Nikolova, 2019) [19] integrate nanotechnology and gene editing. Node size shows the citation count, and the color shows the focus of each publication. The connections show the citation lineage.

Throughout 2000–2025, the top five most cited publications in this field were Caplan (2007) [11], Cui (2010) [12], Schmidt (2003) [13], Banerjee (2009) [14], and Lundborg (2000) [15]. Caplan (2007) was the top-cited publication with 1393 total citations [11,12,13,14,15,16,17,18,19,20]. The work is a review that summarizes the biological basis for the in vivo functioning of MSCs (Figure 6, Table 6).

## 4. Discussion

The pathological process of SCI is complex and can be divided into three stages chronologically: the acute phase, the subacute phase, and the chronic phase. In the acute phase, SCI is primarily characterized by hemorrhage, ischemia, and cell death following injury. In the subacute and chronic phases, factors such as hypoxia, excitatory cytotoxic agents, the generation of free radicals, the disruption of the blood–spinal cord barrier, and the release of proteases further damage neurons. The repair of SCI is a significant clinical challenge, particularly the reconstruction of neural networks. The development of novel tissue engineering scaffolds for the treatment of SCI represents a burgeoning field that has gained remarkable traction over the last two decades [21,22,23]. This study preliminarily presented the research direction of tissue engineering scaffolds in the field of SCI treatment in the past 20 years, attempted to predict research trends, and provided some data for scholars in related fields in the future. With a bibliometric analysis using VOSviewer software [24], the current study overviewed the literature on tissue engineering material-based therapeutic approaches to SCI within the last two decades and generated a visual atlas of the field. Intellectual structures, research trends, main authors, institutions, journals, and countries in this field were analyzed. The current paper shows a panoramic view of the field to young researchers and provides guidance for future research.

With the increasing prevalence of SCI, the demand for innovative treatment strategies has surged [25,26]. The bibliometric analysis revealed a significant growth in research output from 2000 to 2025, with a notable peak in 2019, followed by a plateau, which reflects a confluence of scientific, funding, and collaborative advancements in the field of tissue engineering scaffolds for SCI. The following several factors may explain this trend. Technological breakthroughs: The late 2010s saw pivotal advancements in biomaterial design, such as the optimization of injectable hydrogels and the integration of CRISPR-edited cells into scaffolds. These innovations, validated in preclinical models, spurred a wave of high-impact studies aimed at translating combinatorial therapies. Funding initiatives: Major funding bodies, including the National Natural Science Foundation of China (NSFC) and the NIH, intensified investments in regenerative medicine during this period. For instance, the NSFC’s 2016–2020 funding cycle prioritized spinal cord repair, directly correlating with China’s rising contribution to post-2015 publications. Interdisciplinary Collaborations: The maturation of international consortia and open-access platforms facilitated knowledge exchange, accelerating research productivity. Post-2019 stabilization in publication rates may indicate a transitional phase, where the field shifted from exploratory scaffold design to optimizing delivery mechanisms and addressing translational challenges.

The visual analysis of countries and institutions revealed that the United States and China are the countries with the largest number of articles in this field. The United States sustains leadership in SCI research through sustained National Institutes of Health investments that prioritize translational biomaterial therapies via initiatives like the NIH Blueprint for Neuroscience Research. Elite institutions such as Northwestern University and MIT drive innovation through interdisciplinary hubs—exemplified by Northwestern’s Simpson Querrey Institute integrating materials science and neurobiology to develop injectable hydrogels—while partnerships with biotech firms like 3D Systems accelerate scaffold commercialization. Meanwhile, China’s rapid progress aligns with its 14th Five-Year Plan (2021–2025), designating regenerative medicine as a strategic priority, with National Natural Science Foundation funding doubling since 2015 and targeted grants advancing scaffold development. Talent programs like the Thousand Talents Plan attract global experts such as Dr. Zeng Yuan-shan’s Sun Yat-sen University team, while specialized research centers at Nantong University and Shanghai Jiao Tong University collaborate with state-owned enterprises such as Sinopharm to scale chitosan-based scaffold production. China further diversifies expertise through Belt and Road Initiative partnerships, leveraging Singaporean and Iranian institutions’ niche strengths in nanofiber technologies to complement domestic hydrogel innovations.

From the institutional map, it was found that Northwestern University, the University of Toronto, and Case Western Reserve University are the forefront contributors to high-citation studies, signifying North America’s dominance in this field. Citation per paper analysis showed that the University of Texas has 297.6 citations per paper, followed by Case Western Reserve University with 236 citations per paper, indicating the high research quality of the universities in the field. Meanwhile, the rising influence of Chinese and European institutions illustrates a growing global interest in collaboration, essential for the multidisciplinary approach required in tissue engineering research. The role of collaboration across various institutions and countries is further emphasized by comprehensive network analysis. Such collaboration facilitates the exchange of knowledge, resources, and technology and is crucial for overcoming the inherent complexities of translating scaffold-based treatments from laboratory settings to clinical applications. Scholars newly entering the field and trying to achieve breakthroughs could choose those institutions to study, visit, or work in.

Journal co-citation analysis was carried out to find the major publications in this field and assist scholars in choosing suitable journals. The current study found that *Acta Biomaterialia*, *Journal of Biomedical Materials Research Part A*, *Neural Regeneration Research*, *Tissue Engineering Part A*, and *Advanced Healthcare Materials* are the journals most dedicated to publishing studies on tissue engineering-based therapies for SCI. The field of tissue engineering scaffolds for SCI has been significantly advanced by key researchers such as Stephanie M. Willerth (University of Victoria), who pioneered 3D bio-printed neural tissues and stem cell-laden fibrin scaffolds; Sing Yian Chew (Nanyang Technological University), known for research on aligned nanofiber scaffolds guiding axonal regeneration; Yuan-shan Zeng (Sun Yat-sen University), who integrated traditional Chinese medicine with chitosan hydrogels to reduce glial scarring; Lonnie D. Shea (Northwestern University), a leader in immunomodulatory scaffolds and CRISPR-mediated gene delivery; Jafar Ai (Tehran University), who optimized cost-effective natural polymer scaffolds like alginate–gelatin hybrids; and Somayeh Ebrahimi (University of Tehran), renowned for decellularized spinal cord ECM scaffolds. Collaborative networks—such as Willerth’s partnerships with Case Western Reserve University on gene-edited progenitors, Zeng’s Belt and Road Initiative-driven projects merging TCM with Persian biomaterials, and Shea’s NIH-funded Neuroengineering Consortium—highlight global interdisciplinary efforts. Emerging innovators like Fabian Westhauser (Heidelberg University) have focused on vascularized bioceramic scaffolds, addressing critical gaps in chronic SCI repair. Collectively, these researchers exemplify the shift toward combinatorial therapies (e.g., hydrogels + stem cells + gene editing), bridging foundational discoveries with translational applications, while underscoring the importance of equitable collaboration and scalable solutions to advance personalized, clinically viable treatments for SCI [27,28].

The keyword co-occurrence analysis identified “functional recovery”, “regeneration”, “stem cells”, “hydrogels”, and “transplantation” as key trending topics in the field. This aligns with the overarching goal of not just restoring structural integrity but also fully rehabilitating sensory and motor functions. Innovative approaches, such as the integration of neurotrophic factors and bioactive molecules within scaffolds, could revolutionize treatment efficacy. Cells have the ability to proliferate and differentiate, potentially replacing damaged spinal cord tissue, thus showing promise in the treatment of SCI. Common cell types used in SCI treatment include neural stem cells, bone marrow mesenchymal stem cells (BMSCs), embryonic stem cells, Schwann cells, and olfactory ensheathing cells. However, due to the flow of cerebrospinal fluid and the influence of the microenvironment of the injury site, transplanted cells often fail to effectively colonize the damaged area, resulting in low cell survival rates and unpredictable differentiation. To address these issues, researchers have combined cell transplantation with biomaterials, which has been shown to be highly effective [29]. The advantage of this approach is that biomaterials can serve as scaffolds for cell transplantation, improving or replacing the post-injury environment and providing the physical matrix and nutrients necessary for neural repair. The rise in tissue engineering as a focal point in regenerative medicine highlights its potential to address the complex challenges posed by SCI [30], where hydrogels emerge as pivotal biomaterials mimicking the spinal cord’s extracellular matrix to bridge injury gaps, deliver neurotrophic factors like BDNF, and reduce glial scarring, as demonstrated by thermo-responsive hydrogel success in rat models [31]. Stem cells, particularly mesenchymal and neural progenitors, synergize with scaffolds—such as chitosan composites boosting MSC survival to modulate inflammation and enhance neural repair, though low post-transplantation viability persists as a challenge. Axonal regeneration, hindered by inhibitory factors like Nogo-A, is addressed through aligned nanofiber scaffolds guiding directional growth and ECM-mimicking hydrogels suppressing inhibitory signaling, while functional recovery metrics highlight the efficacy of multimodal strategies combining scaffolds with electrical stimulation [32,33,34].

This study highlights several key strategies employed in tissue engineering, including the development of biomaterials that mimic natural tissue properties, facilitating regeneration and repair. The utilization of stem cells, including mesenchymal and Schwann cells, in conjunction with scaffolds, is instrumental in promoting differentiation and aiding functional recovery post SCI. Despite these advancements, several challenges persist. The intricate nature of the spinal cord microenvironment poses significant obstacles in deploying scaffolds effectively. The risk of immune rejection, infection, and the long-term stability of scaffolds are critical concerns that require further research. Additionally, ensuring consistent functional recovery outcomes across diverse patient demographics remains a challenge.

Ideal biomaterials should possess the following characteristics: (1) injectability, shear thinning, and thixotropy; (2) low biocompatibility, low cytotoxicity, poor immunogenicity, and a lack of mutagenicity; (3) biodegradability; (4) porosity; and (5) the absence of swelling. A wide variety of biomaterials are used for cell transplantation in SCI treatment, primarily divided into natural and synthetic materials [35]. Based on their function and application, they can be categorized into three main types: (1) hydrogels, which can serve as both delivery vehicles and biological scaffolds; (2) microbubbles, primarily used as delivery vehicles; and (3) various biomaterials used to construct biological scaffolds, including collagen, alginate, hyaluronic acid, and nano-supramolecular materials. Below, we discuss the application of these biomaterials in combination with cell transplantation for SCI treatment [35,36,37].

### 4.1. Hydrogels

Hydrogels are characterized by their high water content and mechanical properties similar to collagen in the spinal cord, which is a major structural protein in humans [38]. Therefore, hydrogels can not only serve as carriers for cells and drugs but are also commonly used as scaffolds for cell implantation in the spinal cord. Research has shown that hydrogels are chosen as biomaterials for SCI treatment because they can provide structural scaffolds for axonal and neuronal regeneration, carry cells and active factors, and promote injury repair. Hydrogels can accurately simulate the body’s environment and the central nervous system [39] and possess several excellent properties: (1) good biocompatibility, avoiding immune suppression reactions; (2) excellent physical and chemical stability, effectively providing conditions for cell attachment and proliferation; (3) appropriate porosity and permeability for the metabolism of nutrients and waste; and (4) good biodegradability. Additionally, after being injected into the local injury site, hydrogels can fill tissue defects, promote tissue repair, and cause no invasive damage, ensuring high safety.

### 4.2. Microspheres

Recent studies on microspheres have focused on their use as drug carriers for targeted delivery, enabling integrated diagnosis and treatment. Under ultrasound, microspheres can create pores in cell membranes, allowing drugs to enter cells more effectively and achieve targeted therapy [40].

### 4.3. Bioengineered Scaffolds

The early application of bioengineered scaffolds in SCI treatment can correct the pathophysiological state of the spinal cord and reduce the extent of secondary injury. During the stable phase, bioengineered scaffolds combined with stem cell transplantation and cytokine induction can help promote the recovery of spinal cord function [41]. Therefore, bioengineered scaffolds have become a key component of cell transplantation for SCI treatment. Currently, there are five main biomaterials used to construct bioengineered scaffolds.

#### 4.3.1. Collagen

Collagen is an important component of the extracellular matrix in the spinal cord and has cell adhesion properties, making it widely used as a scaffold to promote stem cell adhesion, differentiation, and migration [42]. Collagen scaffolds are unique biomaterials for SCI repair due to their excellent biocompatibility, acceptable biodegradability, and low antigenicity. Additionally, at the SCI site, collagen can carry growth factors, regulate the local microenvironment, reduce scar formation, and facilitate injury recovery. Cholas et al. [43] found that implanting a type I collagen scaffold alone in a rat SCI model reduced the number of macrophages and angiogenesis on the scaffold after four weeks, laying the foundation for enhancing the regenerative response in SCI.

#### 4.3.2. Chitosan

Chitosan is a natural polysaccharide with biocompatibility, non-toxicity, antibacterial properties, and biodegradability. It also promotes cell attachment and growth, making it an ideal scaffold material [44]. However, it has the disadvantages of high swelling and rapid degradation, so it is often combined with other materials when used in SCI treatment.

#### 4.3.3. Alginate

Alginate is a hydrophilic linear polysaccharide naturally found in the cell walls of seaweed. Due to its unique chemical structure and properties, alginate is often used as an adhesive or stabilizer in SCI treatment [45]. Alginate scaffolds also serve as suitable reservoirs for BMSCs.

#### 4.3.4. Hyaluronic Acid

Hyaluronic acid is a natural polymer widely studied for scaffold production. As a component of the extracellular matrix, hyaluronic acid is biocompatible and interacts with various cell receptors, coordinating cell communication and behavior. Importantly, hyaluronic acid can undergo various simple chemical modifications, allowing cross-linking between polymer chains and the formation of highly tunable scaffolds [46]. For stem cell delivery applications, hyaluronic acid-based hydrogels are tunable, enabling the design and customization of microenvironments that influence stem cell fate and provide protection for transplanted cells. Small-molecule drugs can be delivered directly through hyaluronic acid-based hydrogels to elicit the desired therapeutic response. Biomolecules can be tethered to hyaluronic acid polymer chains or embedded in hydrogels for local delivery. Elliot Donaghue et al. [47] demonstrated that hyaluronic acid-based hydrogels delivering NT-3 promoted axonal regeneration and improved motor function recovery in rats after SCI.

#### 4.3.5. Nano-Supramolecular Materials

Nano-supramolecular materials with fine structures can interact with cell surface receptors and components, providing strong conditions for remodeling the cellular microenvironment. With advancements in tissue engineering and genetic engineering, medical nano-supramolecular materials used as spinal cord tissue engineering scaffolds not only improve cell–material adhesion, biocompatibility, and biodegradability but also facilitate cell differentiation and proliferation. Combining genetic engineering with the advantages of nano-biomaterials could offer a new pathway for the clinical treatment of SCI [48]. Sun et al. [49] prepared ladder-shaped nanofiber neural conduits and implanted them into rats with complete spinal cord transection. The experiments showed that both implants effectively reduced scar formation and inflammation, restored neural stem cells, and increased nerve fiber growth by inhibiting cell infiltration and accumulation in scaffolds. Notably, nanofiber neural conduits further promoted guided axonal extension, providing a favorable microenvironment for neural regeneration after SCI. Li et al. [50] reported that combining nano-hydrogels with human adipose-derived stem cells modulated the inflammatory microenvironment, protected neurons and axons, and promoted motor function recovery in rats with severe SCI.

Despite significant advancements in tissue engineering scaffolds for SCI, clinical translation faces barriers including regulatory complexities for combination products, safety concerns such as immunogenicity and long-term biocompatibility, manufacturing challenges in scalability and sterilization, and high costs limiting accessibility. To address these hurdles, strategies such as modular scaffold designs for regulatory compliance, AI-driven manufacturing for reproducibility, global preclinical testing standards, public–private partnerships for funding, and equitable access frameworks using low-cost biomaterials are essential. Emerging technologies like smart scaffolds with biosensing capabilities and machine learning-optimized designs, coupled with interdisciplinary collaboration and adaptive regulatory frameworks, hold promise for transforming these innovative therapies from experimental tools to mainstream treatments, ultimately improving outcomes for SCI patients worldwide.

Looking forward, there is a pressing need for continued interdisciplinary research, combining insights from neuroscience, materials science, and biotechnology. Future trajectories emphasize combinatorial therapies, such as CRISPR-edited stem cells in hydrogels for sustained growth factor release, alongside bioengineered microenvironments using organ-on-a-chip platforms to personalize scaffold testing with patient-derived cells. Ethically driven precision approaches, including iPSC-derived neural progenitors in tailored scaffolds, and AI-driven smart materials (e.g., pH-sensitive hydrogels) signal a paradigm shift toward adaptive, patient-centric therapies poised to overcome SCI’s historical barriers [36].

## 5. Conclusions

This study underscores the rapid advancement and growing interest in tissue engineering for SCI treatments over the past twenty years. While significant progress has been made in developing effective scaffolds, ongoing research is crucial to address existing challenges and harness emerging trends for improved patient outcomes. As the field continues to evolve, it holds promising potential to transform SCI treatment paradigms, ultimately improving the quality of life of affected individuals.

## Figures and Tables

**Table 1:** Main countries contributing to research on tissue engineering-based treatment strategies for spinal cord injury.

Countries	Documents	Citations	Total Link Strength
USA	370	20,956	2356
China	259	5854	1725
Canada	67	3423	374
England	45	2478	474
Italy	45	1988	302
Germany	70	1969	323
Australia	35	1519	406
Singapore	29	1386	483
Iran	60	1323	18
Japan	27	1121	137

**Table 2:** Main institutions that have contributed the greatest number of papers to research on tissue engineering-based treatment strategies for spinal cord injury.

Institutions	Documents	Citations	Citation per Paper	Total Link Strength
Northwestern University	20	2342	117.1	149
University OF Toronto	26	2146	82.5	286
Case Western Reserve University	7	1652	236.0	11
University Texas	5	1488	297.6	96
Washington University	17	1396	82.1	235
University of Pennsylvania	22	1124	51.1	150
Massachusetts Institute of Technology	13	1041	80.1	121
Harvard University	19	853	44.9	61
University Milano Bicocca	7	782	111.7	117
University California Berkeley	6	774	129.0	49

**Table 3:** The most popular key words in studies on tissue engineering-based treatment strategies for spinal cord injury.

Key Words	Occurrences	Total Link Strength
Functional recovery	194	2948
In vitro	182	1776
Regeneration	181	1779
Transplantation	149	1514
Differentiation	130	1207
Axonal regeneration	124	1378
Stem cells	118	1119
Schwann cells	105	1100
Extracellular matric	90	877
Hydrogel	83	853

**Table 4:** Journals with the highest number of publications in terms of research on tissue engineering-based treatment strategies for spinal cord injury.

Publications	Documents	Citations	Total Link Strength
*Biomaterials*	55	4112	590
*Acta Biomaterialia*	35	1745	298
*Journal of Biomedical Materials Research Part A*	33	1340	298
*Neural Regeneration Research*	20	219	152
*Tissue Engineering Part A*	16	631	151
*Advanced Healthcare Materials*	14	543	147
*Acs Biomaterials Science & Engineering*	14	465	147
*Journal of Materials Chemistry B*	12	586	130
*Journal of Neurotrauma*	8	213	128
*Advanced Functional Materials*	7	399	116

**Table 5:** Top 10 most productive authors in research on tissue engineering-based treatment strategies for spinal cord injury.

Author	Documents	Citations	Total Link Strength
Ai, Jafar	19	396	10,891
Zeng, Yuan-shan	16	518	28,469
Ebrahimi Somayeh	15	295	10,173
Chew, Sing yian	14	687	24,293
Shea, Lonnie d.	14	769	19,786
Willerth, Stephanie m.	13	865	15,017
Westhauser, Fabian	13	303	7820
Moghaddam, Arash	13	219	7439
Zeng, Xiang	12	381	22,083
Rossi, Filippo	12	281	11,612

**Table 6:** Top cited publications in the research of tissue engineering based treatment strategies for spinal cord injury.

Document	Citations	Links
Caplan (2007) [11]	1393	1
Cui (2010) [12]	1115	7
Schmidt (2003) [13]	902	59
Banerjee (2009) [14]	463	20
Lundborg (2000) [15]	450	6
Beachley (2010) [16]	417	0
Liu (1999) [17]	402	13
Parr (2007) [18]	373	12
Nikolova (2019) [19]	316	2
Evans (2001) [20]	311	12

## Data Availability

Data and materials are available from the corresponding author upon request.

## Abbreviations

The following abbreviations are used in this manuscript:

SCI	Spinal cord injury
WoS	Web of Science
NSFC	National Natural Science Foundation of China
LD	National Institutes of Health
BMSCs	Bone marrow mesenchymal stem cells
